# Complete mitochondrial genome of endangered Arabian tahr (*Arabitragus jayakari)* and phylogenetic placement

**DOI:** 10.1080/23802359.2022.2090295

**Published:** 2022-06-28

**Authors:** Ahmed N. Al-Rawahi, Zahir S. Alalawi, Sajjad Asaf, Abdul Latif Khan, Adil Khan, Haitham Al-Rawahi, Steven Ross, Mansoor Al-Jahdhami, Ahmed Al-Harrasi

**Affiliations:** a Natural and Medical Sciences Research Center, University of Nizwa, Nizwa, Oman; b Office for Conservation of the Environment, Diwan of Royal Court, Sultanate of Oman, Muscat, Oman; c Department of Biotechnology, College of Technology, University of Houston, Houston, TX, USA; d Keefer Ecological Services Ltd., Cranbrook, Canada

**Keywords:** Arabian Tahr, endangered, mitogenome, Red list

## Abstract

The Arabian tahr (*Arabitragus jayakari*) endemic to mountains of northern Oman and the United Arab Emirates, however, the species is faced with significant threats to its population. Because of its small and dwindling population, it is listed as Endangered. Here, we sequenced and assembled the mitochondrial (mt) genome of *A. jayakari* into 16,485 bp with 39.6% GC content. It also contains 13 protein-coding genes, 22 tRNA genes, and two rRNA genes. Phylogenetic analysis of *A. jayakari* with related 12 species mt genomes showed that *A. jayakari* forms a monophyletic clade with *Hemitragus jayakri*. In the current context of a changing environment, evolutionary analysis based on mitochondrial genome will aid in identifying evolutionary changes among different species and analyzing shared gene pools to counteract threats.

The Arabian tahr (*Arabitragus jayakari*_Thomas 1894*)* is a rare mountainous ungulate species found only in the 700 Km Hajar Mountain chain of Oman and the United Arab Emirates, where it prefers rugged terrains of steep mountains (Ross et al. 2019). Recent assessment of tahr across the entire range concluded with a population estimate of 2,446 individuals left in wild (Ross et al. 2019). Therefore, it was assessed to be in the category of endangered species according to the Red List Criteria of the International Union for Conservation of Nature (IUCN). Tahr is facing many threats imposed by habitat destruction and fragmentation (Insall 2008) sourced mainly via mineral mining, industrial and urban development as well as pouching, competition, and diseases transmission (Ross et al. 2019). Environmental stress associated with lower oxygen pressure, freezing temperatures, increased UV radiation, steep slopes, and lower availability of food sources may have put major selective pressures on the development of the mitochondrial genome in organisms living at high altitudes (Ropiquet and Hassanin 2005). Hassanin et al. (2009) used polymerase chain reaction (PCR) to sequence the mitochondrial genome of Tahr using overlapping fragments. However, in this study, we used the Ion S5 next-generation sequencing technology to sequence the complete mitochondrial genome of *A*. *jayakari* for the first time and compared it to related species to determine the phylogenetic relationship.

During veterinary routine checkup each individual tahr was manually restrained and the blood samples were collected in Omani Captive Breeding Center for Mammals at Bait al Barakah (23.7093° N, 58.0911° E) in Muscat, Oman. The Omani Wild Animals Breeding Center, Royal Court Affairs, Muscat, Oman, approved the sample collection, which was done in accordance with the regulations of the International Union for Conservation of Nature (IUCN). The sample (specimen voucher: UoN_00012) was deposited in the Museum of Conservation of the Environment, Diwan of Royal Court, for more information about this voucher please contact Ahmed N. Al-Rawahi, email: ahmed.alrawahi@unizwa.edu.om. Total genomic DNA was extracted using a QIAGEN DNeasy Blood & Tissue Kit. This was quantified using Qubit 3.0 with high sensitivity kit, followed by gel electrophoresis. The high-quality DNA was used for library preparation according to the manufacturer's instructions (Life Technologies USA, Eugene, OR, USA). The DNA was enzymatically fragmented into 400 bp using the Ion ShearTM Plus Reagents kit, and libraries were constructed with the Ion XpressTM Plus gDNA Fragment Library kit. After library preparation, the Ion OneTouchTM 2 instrument was used to amplify the template, and the amplified templates were enriched (Ion OneTouchTM ES enrichment system) with Ion 530 & 520 OT2 Reagents. The samples were loaded onto the Ion S5 530 Chip using the Ion S5 sequencing protocol. Trimmomatic (Bolger et al. 2014) was used to trim raw reads, and Geneious Prime 2019.3.1 was used to map them to the *Hemitragus jayakari* (NC020621) mitogenome (Biomatters Ltd., Auckland, New Zealand). To undertake structural and functional annotation, web servers (MITOS) (Bernt et al. 2013), and GeSeq (Tillich et al. 2017) were used. Following that, homology searches on GenBank and manual curation were used to confirm that the annotated genes were accurate. Finally, mtDNA sequences were aligned and a phylogenetic tree was constructed using the program ClustalW implemented in MEGAX.

The A. *jayakari* (MN971587) mitochondrial genome is 16,485 bp long and contains 13 protein-coding genes, two ribosomal RNA genes (rRNA), 22 transfer RNA genes (tRNA), and a control region (D-loop). The protein-coding genes were found in a range of 201 bp (*atp*8) to 1,830 bp (nad5). They included 13 genes for the production of ATP synthase and the electron transport chain, which is made up of the following subunits: 7 complexes I subunits (nad1,2,3,4,5,6 and nad4L), 1 complex III subunits (*cob*), 3 complex IV subunits (*cox*1-3), and 2 complex V subunits (*atp*6 and *atp*8). The GC contents of whole genome, protein-coding genes, transfer RNA and ribosomal RNA were 39.6%, 39.9%, 36.4% and 40.3%, respectively. The mitochondrial genome of *A. jayakari* contains an A + T bias with an overall nucleotide composition of A = 5,587 (33.9%), T = 4,369 (26.5%), C = 4,403 (26.7%), and G = 2,126 (12.9%). The A + T content of the mitogenome is 60.4%. Protein coding regions, ribosomal RNA and transfer RNA accounted for 69.02%, 15.3% and 9%, respectively. A total of 50 repeats [including forward repeats (23), palindromic repeats (11) and reverse repeats (16)] were found in *A. jayakari* mitochondrial genome.

Phylogenetic analysis revealed that the mitochondrial genome of *A. jayakari* forms a monophyletic clade with *Hemitragus jayakri* (NC020621) and *Ammotragus lervia* (NC009510) (Figure 1). To sum up, this study provides the information of *A. jayakari* mitochondrial genome, which will also be necessary for further taxonomic classification, phylogenetic reconstruction and implementing conservation strategies.

**Figure 1.**
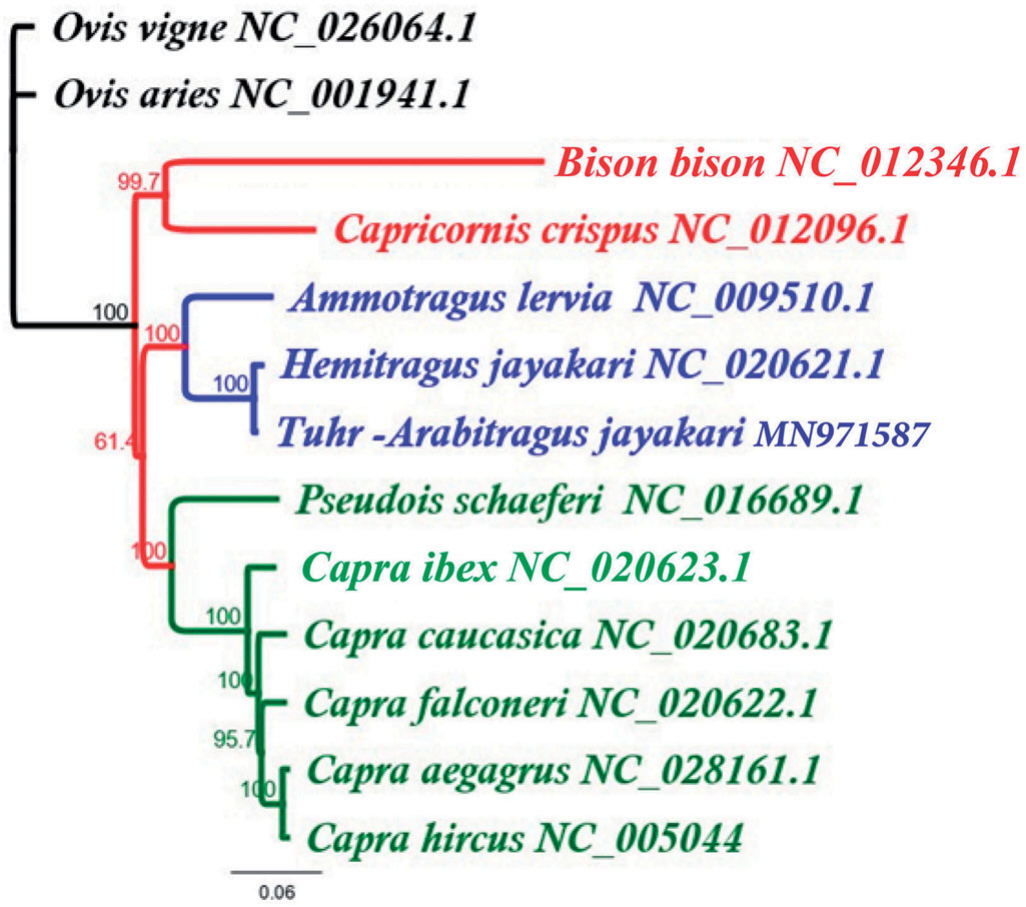
Maximum Likelihood phylogeny of *A. jayakari* based on complete genome. Bootstrap values are indicated at each node.

## Data Availability

The data supporting this study’s findings are openly available in GenBank of NCBI at https://www.ncbi.nlm.nih.gov/nuccore/MN971587, accession number MN971587. The associated BioProject, SRA, and Bio-Sample numbers are PRJNA807824, SRR18055241, and SAMN26001840 respectively.
